# Supplementary material for: Impact of visual callosal pathway is dependent upon ipsilateral thalamus
Source: Nat Commun. 2020 Apr 20;11:1889. doi: 10.1038/s41467-020-15672-4 (PMC7171107; doi:10.1038/s41467-020-15672-4)
Supplement: Supplementary file 1 — Supplementary Information [file 41467_2020_15672_MOESM1_ESM.pdf]

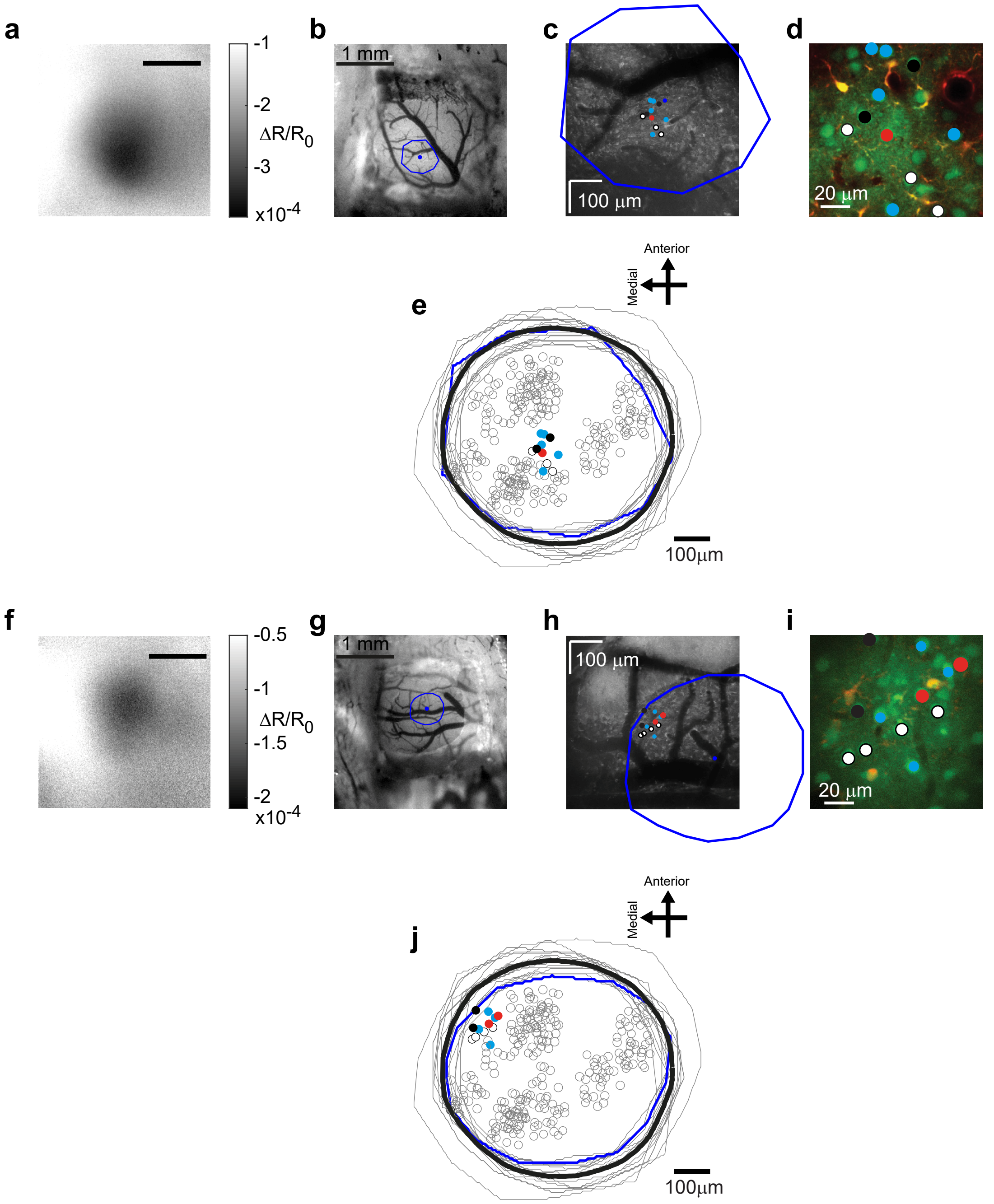

Supplementary Figure 1. Alignment of multiphoton data using intrinsic optical signal imaging response.

a, example intrinsic optical signal imaging response from one animal evoked by grating stimuli in front of the animals nose. Scale bar as in **b**. **b**, image of cortical surface vasculature at the location where the response in **a** was recorded. Blue line indicates the 90th percentile of the intrinsic optical imaging response in **a**. Blue spot represents the contours center of mass. **c**, matching surface blood vessels in multiphoton imaging data to those within the blue contour in **b**. Blue contour is a scaled version of that shown in **b**, aligned to the multiphoton image using the location of its center of mass (blue spot) with respect to the blood vessels in the image. Turquoise, black, red and white-filled black circles denote the location of the matching neurons shown in **d**. **d**, multiphoton overview image in layer 2/3 showing a field of view from which visual responses were acquired. OGB loaded neurons shown in green, SR101 counterstained astrocytes in red, OGB loaded astrocytes appear yellow. Field of view same as that shown in Figure 1**e**,**h**. **e**, same data as presented in Figure 1**i** with the cells shown in **c** and **d** shown colored and all other neurons shown as open grey circles. **f**, **g**, **h**, **i** and **j** are examples from a second dataset from another animal, with panel conventions as for **a**, **b**, **c**, **d** and **e** respectively.

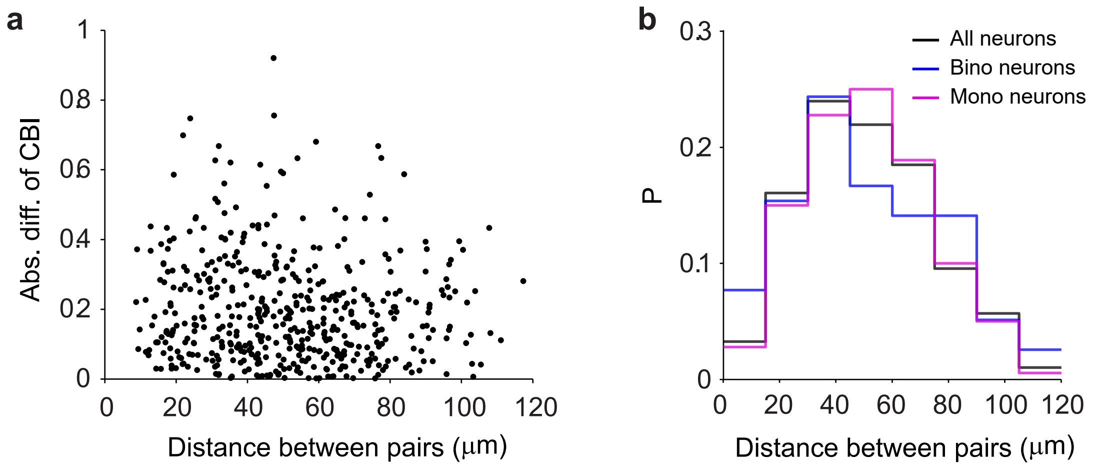

Supplementary Figure 2. Monocular and binocular neurons are interspersed and do not cluster.

a, scatter plot of the distances between neuronal pairs and differences in their contralateral bias indices. Correlation was not significant (Pearson's rho = -0.079, P = 0.082). **b**, distribution of pairwise distances for all neuronal pairs, binocular-neuron pairs and monocular-neuron pairs. The distributions were not significantly different (all neuronal pairs vs binocular-neuron pairs, 2-sided KS-test P = 0.457; all neuronal pairs vs monocular-neuron pairs, 2-sided KS-test P = 0.999).

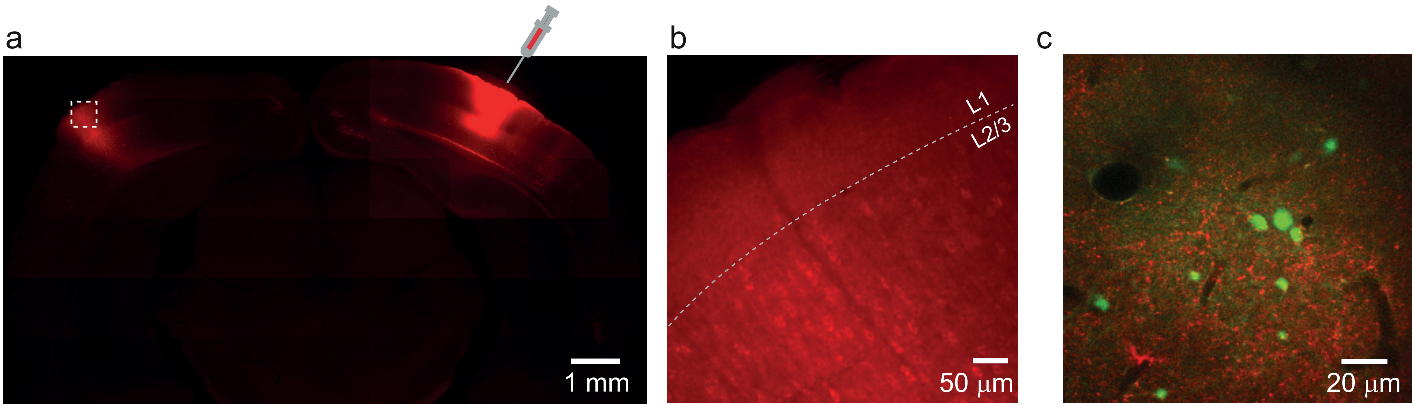

Supplementary Figure 3. Lack of cholera toxin B retrograde labelled neurons in layer 1.

a, coronal section from one animal in which neurons in the primary visual cortex (V1) had been retrogradely labelled by an injection of Alexa 594-conjugated cholera toxin B into contralateral V1 (location denoted by syringe schematic). **b**, enlarged image of the superficial cortical region outlined by the dashed box in **a** in V1 contralateral to the injection site. Retrogradely labelled neuronal somata are visible in layer 2/3, but not in layer 1. **c**, overview 2-photon image from layer 1 in an animal in which neurons had been labelled with OGB-1 (green) and callosally-projecting neurons retrogradely labelled with cholera toxin B.

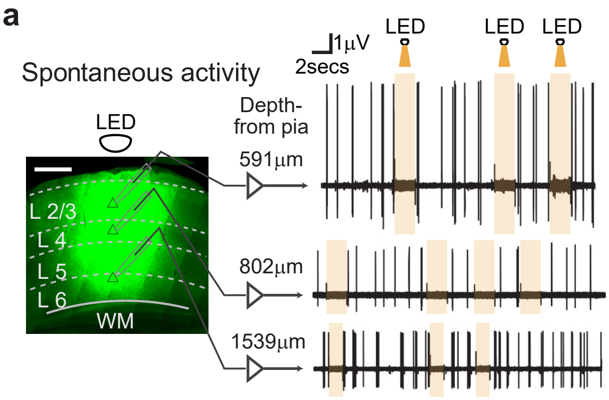

Supplementary Figure 4. eArchT enabled fast and reversible inactivation of neurons from cortical layer 2 to 6.

Examples of cell-attached eletrophysiological recordings showing optogenetic inactivation (yellow boxes) of neurons in vivo at the depths indicated (scale bar in left panel 0.5 mm).

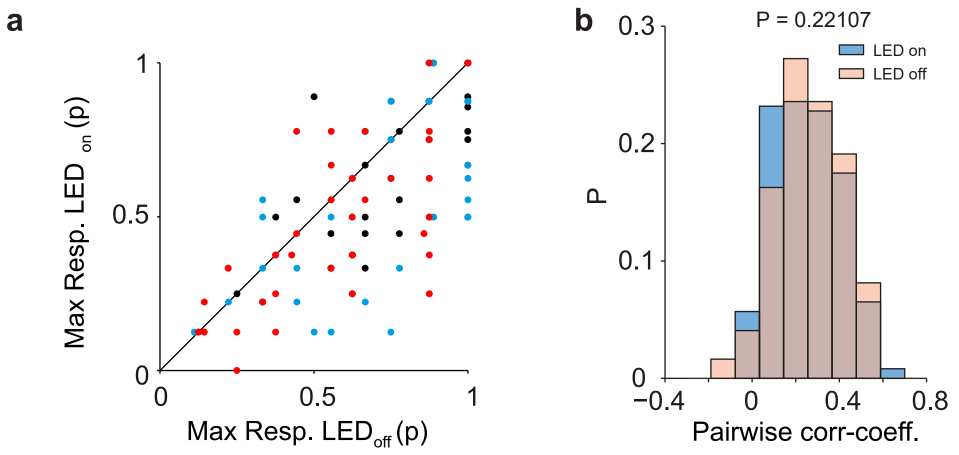

Supplementary Figure 5. Preferred orientation response and pairwise correlations during vc pathway inactivation.

a, comparison of maximum response probabilities at the preferred orientation (maximum stimulus-evoked response) from the same populations of neurons as in Fig. 3**e** (N = 6 populations) during binocular (black), contralateral (turquoise) and ipsilateral stimulation (red) in control (LED off) and with eArchT inactivation of contralateral visual cortex (LED on). **b**, distribution of pairwise correlations in neuronal activity before (orange) and after (blue) vc pathway inactivation. The two distributions were not statistically different (2-sided KS-test P = 0.221). Note that cases where the blue bars are hidden behind the orange bars are indicated by slightly darker orange colour.

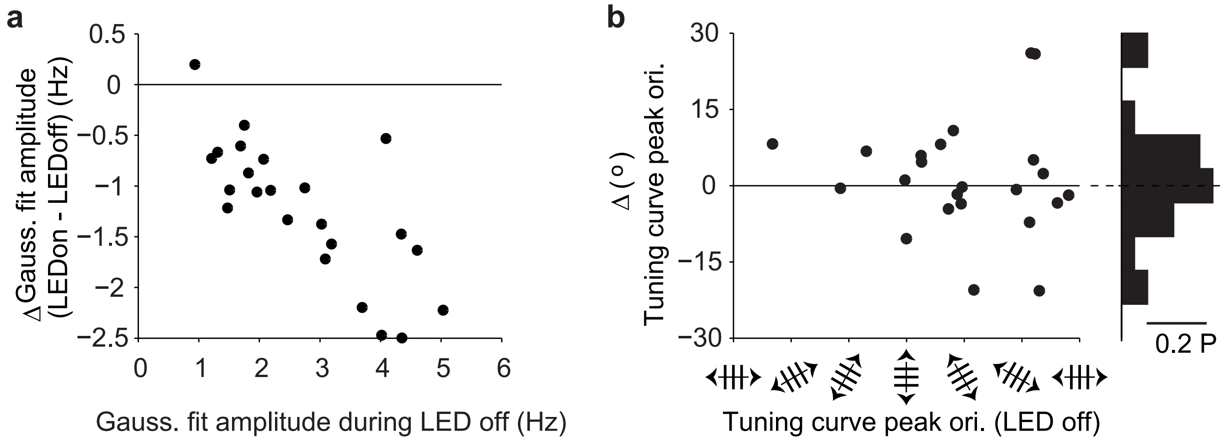

Supplementary Figure 6. Vc pathway inactivation reduced the response amplitude of neurons, but did not change their preferred orientation.

a, change in peak amplitude of Gaussian tuning curve fitted to neuronal spiking response in control versus during vc pathway inactivation as a function of peak Gaussian amplitude in control. Data shown for all eye condition responses where vc pathway inactivation caused a significant change in response amplitude (green dots in Supplementary Fig. 7 and 8). Cases where visual responses were reduced to be not significantly different to spontaneous activity not included (orange dots in Supplementary Fig. 7 and 8). **b**, change in the preferred orientation (orientation at the peak of the Gaussian tuning curve fitted to neuronal spiking response) as a function of orientation at the peak of the fitted Gaussian in control.

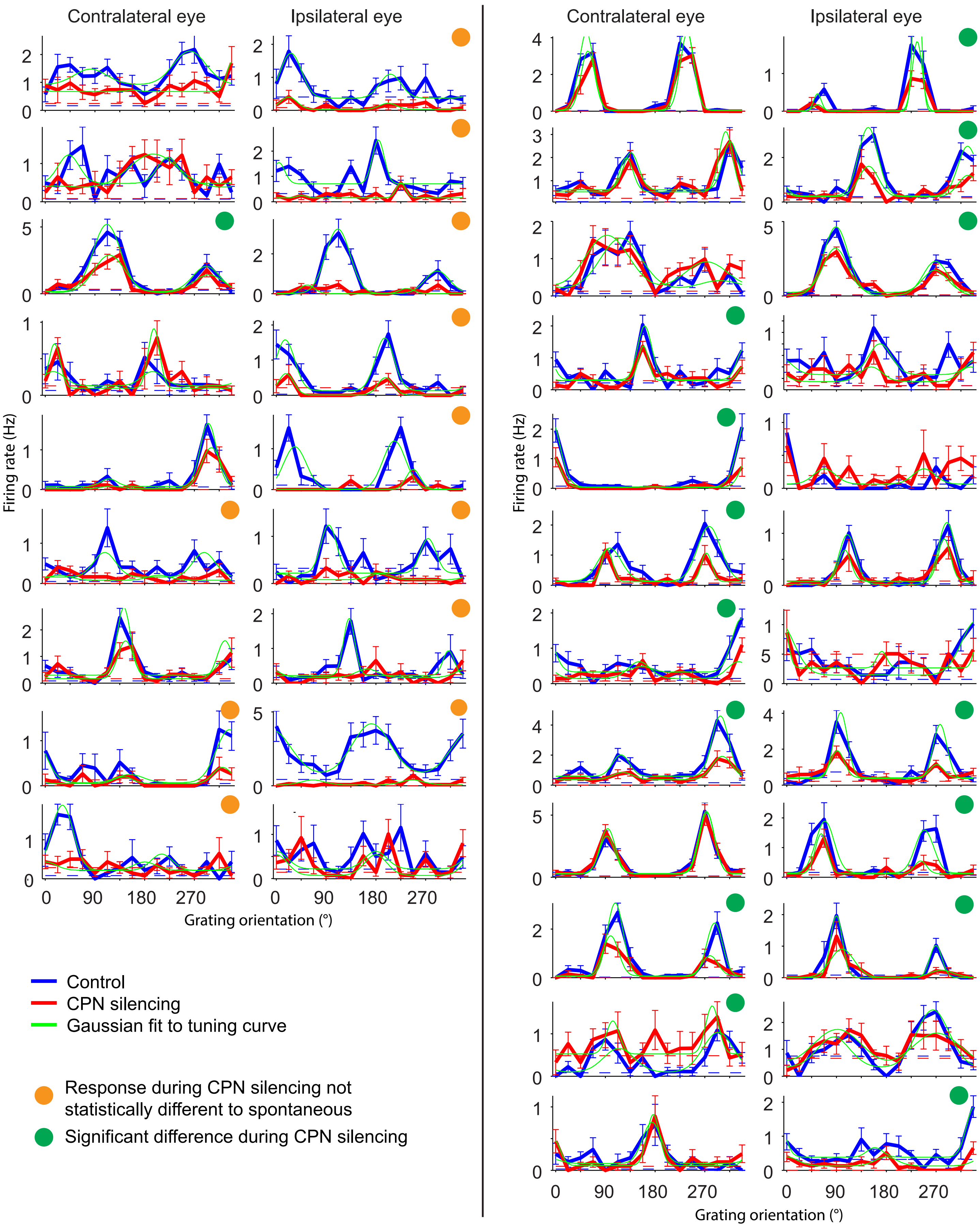

Supplementary Figure 7. Gaussian tuning curve fitted to spiking response of binocular neurons.

Tuning curves for all binocular neurons that showed a significant change upon vc inactivation. Shown are orientation responses in control (blue) and during vc inactivation (red), and the Gaussian fits to the orientation response data (light green). Orientation responses are mean±SEM. Significant difference to control conditions denoted by green dots and complete cessation of visually induced responses (response not significantly different from spontaneous spiking) denoted by orange dots. X-axes show grating orientation in degrees, y-axes show spiking rate in Hz. Spontaneous spiking for the different conditions is indicated by dashed lines.

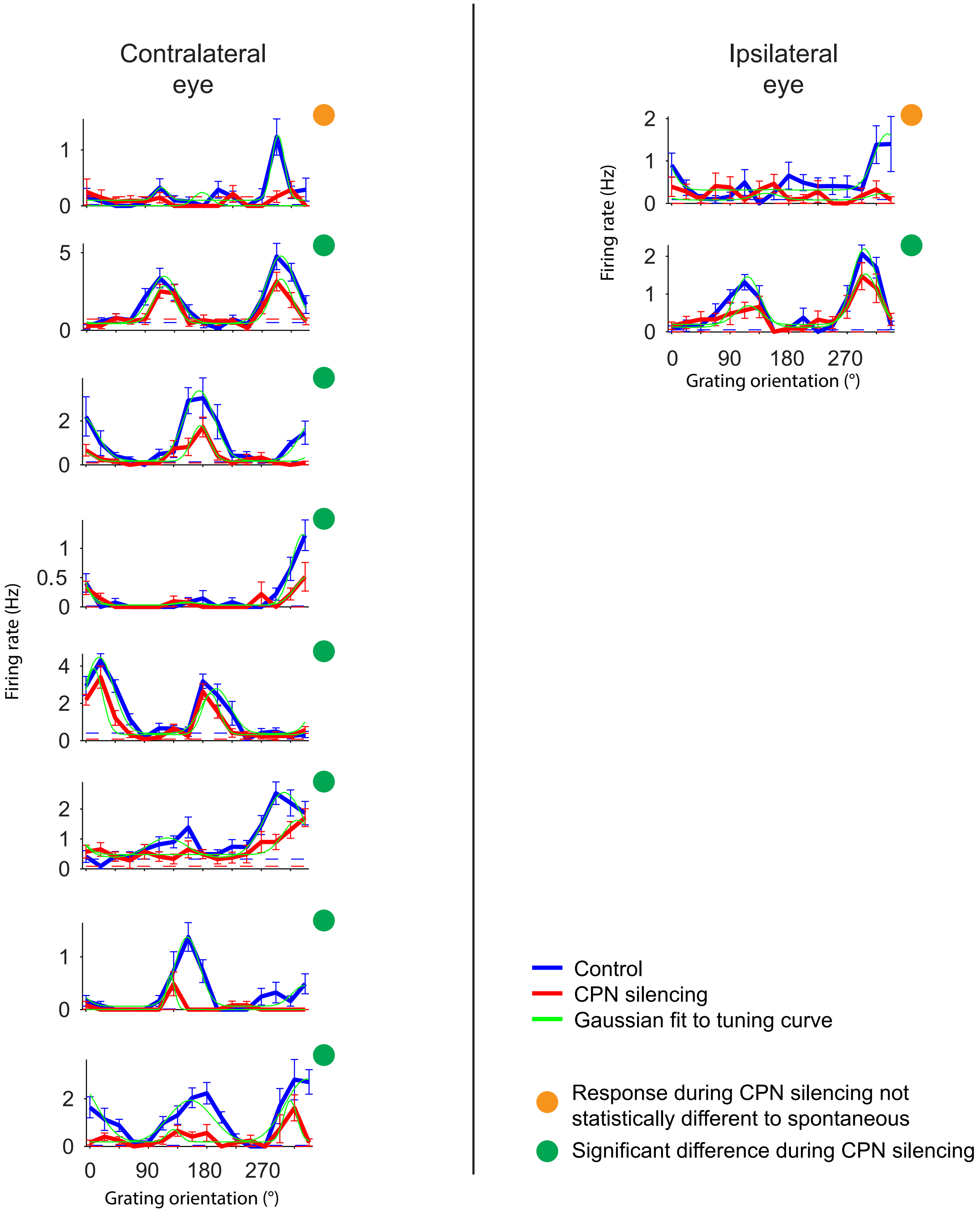

Supplementary Figure 8. Gaussian tuning curve fitted to spiking response of monocular neurons.

Tuning curves for all monocular neurons that showed a significant change upon vc inactivation. Shown are orientation responses in control (blue) and during vc inactivation (red), and the Gaussian fits to the orientation response data (light green). Orientation responses are mean±SEM. Significant difference to control conditions denoted by green dots and complete cessation of visually induced responses (response not significantly different from spontaneous spiking) denoted by orange dots. X-axes show grating orientation in degrees, y-axes show spiking rate in Hz. Spontaneous spiking for the different conditions is indicated by dashed lines.

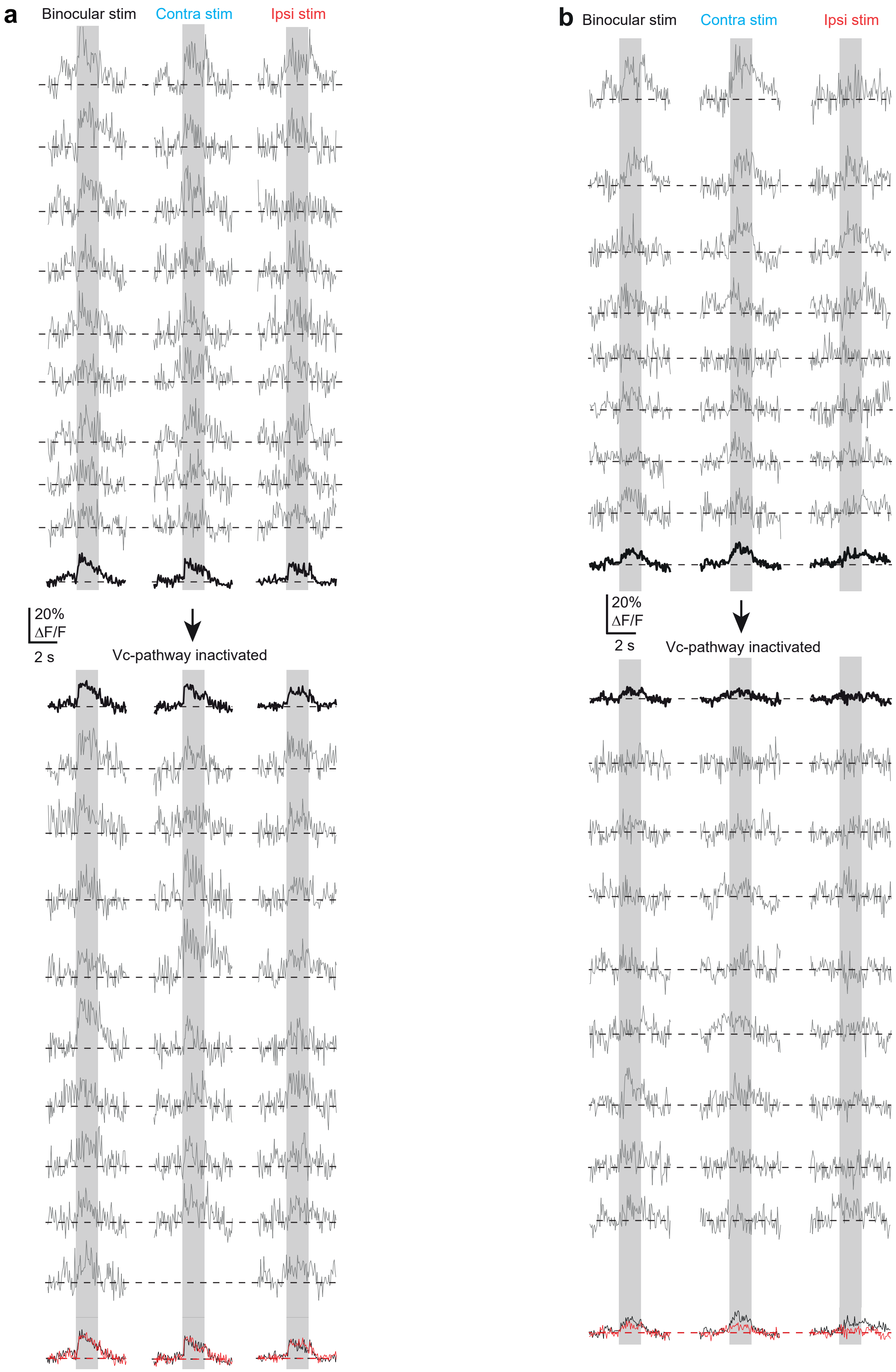

Supplementary Figure 9. Detail of example responses from figure 4b,c.

a, same data as shown in Figure 4**b**, but with individual traces (grey) separated to illustrate visual responsiveness, for control and vc-pathway inactivated conditions as indicated. Average traces are shown in black, with an overlay of control (black) and after vc-pathway inactivation (red) shown at bottom. **b**, same data as in Figure 4**c**, with traces separated as described above.

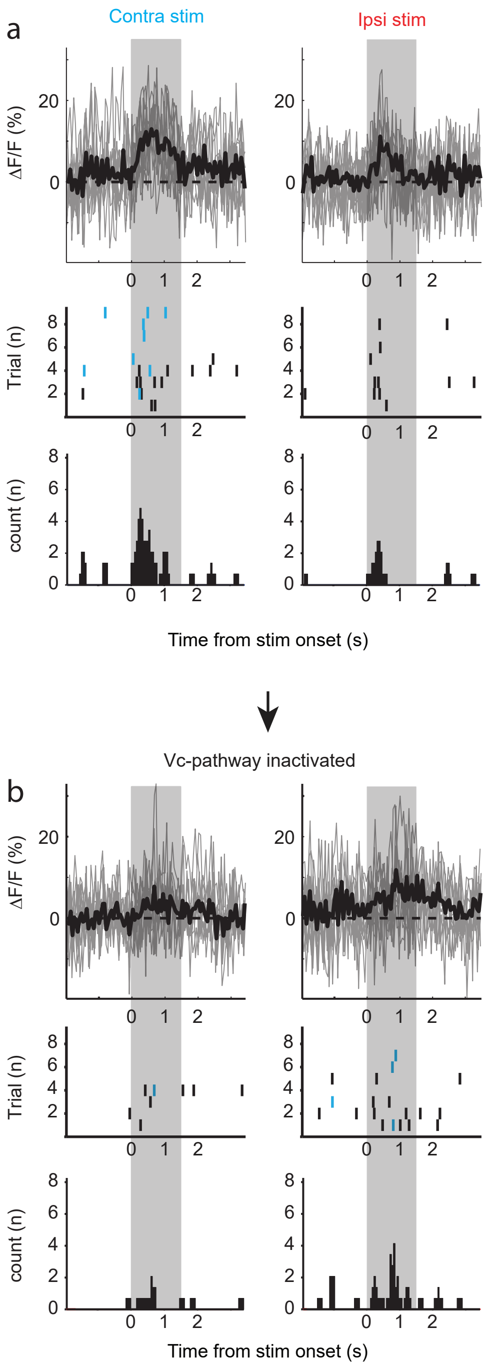

Supplementary Figure 10. Example neuron showing strong reduction in contralateral visual responsiveness on vc pathway inactivation with lesser effect on ipsilateral responsiveness.

a, example Ca^{2+} kinetics (upper panels), inferred spike raster plots for each stimulus trial (middle) and PSTH (lower) for one neuron in response to contralateral (left) and ipsilateral (right) eye stimulation. The Ca^{2+} kinetic panels show individual responses (grey) and average response (black). Ticks in raster plots denote one (black) or two (light blue) inferred spikes per imaging frame. **b**, example data from the same neuron as in panel a but for visual stimuli during vc pathway inactivation. Panel and labelling conventions as in **a**.

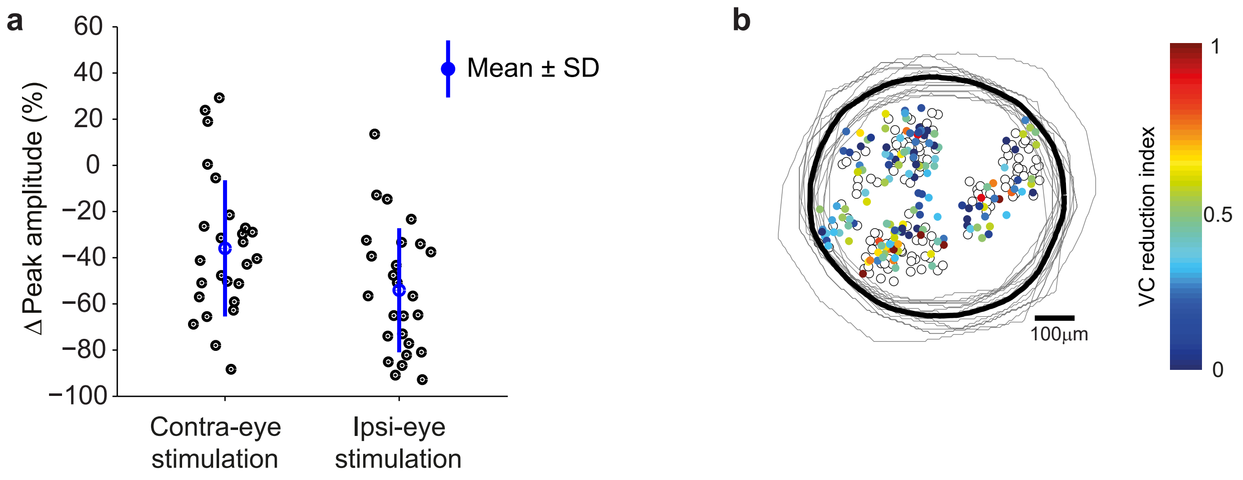

Supplementary Figure 11. Comparison of the effect of vc pathway inactivation from ipsilateral crossed and contralateral uncrossed pathway in binocular neurons and lack of clustering of neurons whose responses were modulated by vc pathway inactivation.

a, change in peak amplitude of spiking response of binocular neurons during contra-eye stimulation and ipsi-eye stimulation (26 neurons from 13 animals). The change during ipsi-eye stimulation was larger compared to change during contra-eye stimulation (2-sided Mann-Whitney U test P = 0.036). **b**, map of neuron locations color-coded by vc reduction index. Map created as for that shown in Fig. 1**i**. No clustering of neurons according to the extent to which responses were modulated by vc-pathway inactivation was observed.

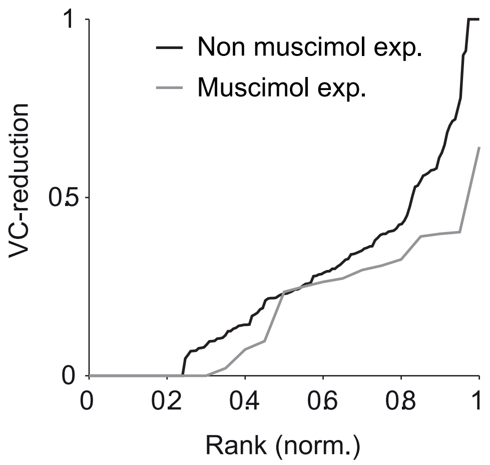

Supplementary Figure 12. Comparable effect of vc pathway inactivation during non-muscimol experiments and muscimol experiments.

The effect on neuronal responses of inactivation of vc pathway during non-muscimol and muscimol experiments, with neurons in each group ranked based on the magnitude of their activity reduction. The progression of effect across neurons was not different between the two experimental groups (2-sided KS-test P = 0.4741).

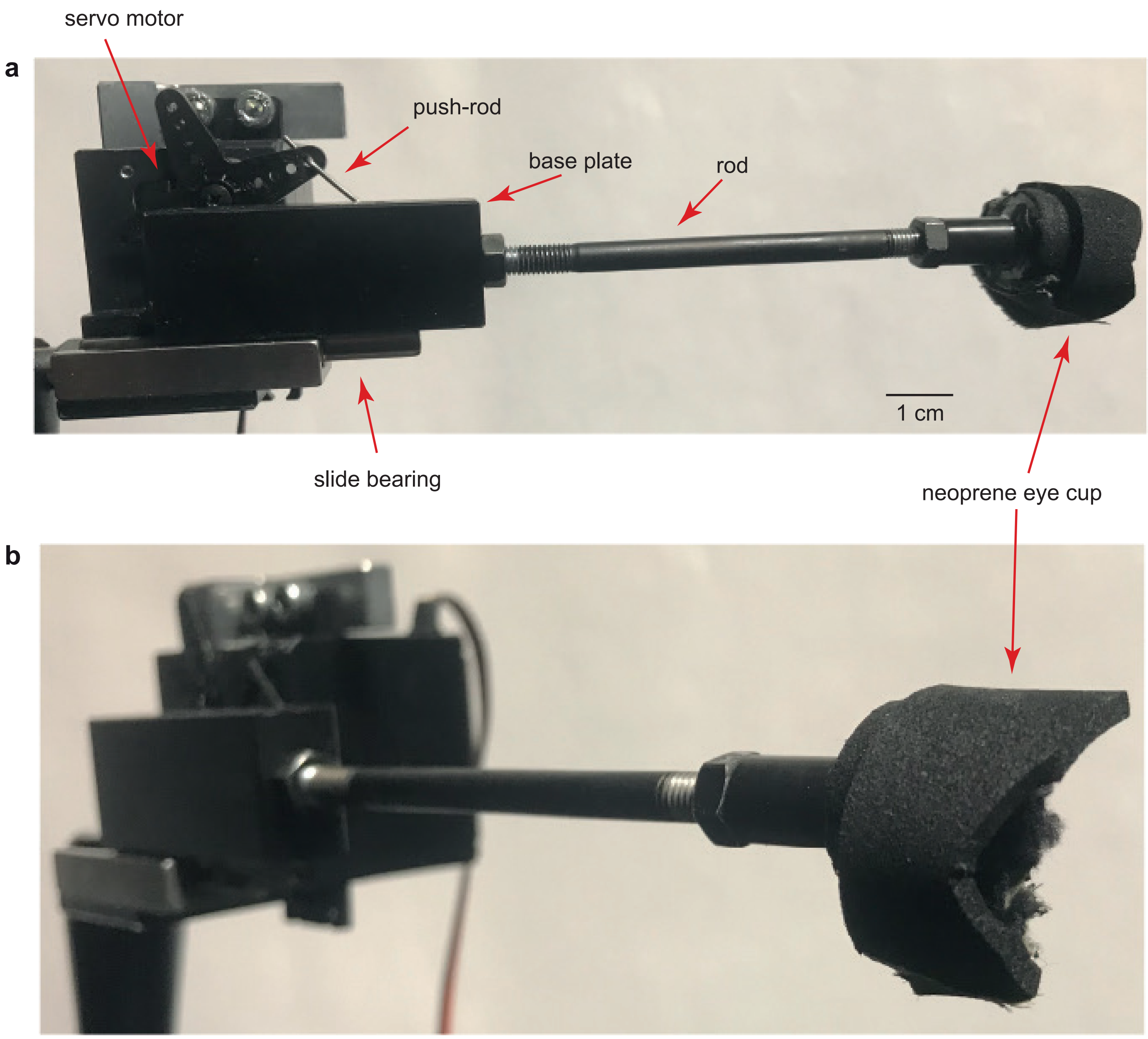

Supplementary Figure 13. Automated eye shutter.

a, side view of one custom-made automated shutter used for preventing visual stimulus presentation to one eye. Shutter consists of a neoprene eye cup, which was placed over the eye, connected to a servo motor, which enabled the eye cup to be withdrawn away from the eye allowing visual stimulus presentation. The eye cup was attached to a rod and base plate, which was connected to the servo motor by a stiff push-rod, moved smoothly forward and backward on a slide bearing. **b**, semi-frontal view of eye cup.
