# Supplementary material for: Impact of visual callosal pathway is dependent upon ipsilateral thalamus
Source: Nat Commun. 2020 Apr 20;11:1889. doi: 10.1038/s41467-020-15672-4 (PMC7171107; doi:10.1038/s41467-020-15672-4)
Supplement: Supplementary file 3 — Reporting Summary [file 41467_2020_15672_MOESM3_ESM.pdf]

nature research

Corresponding author(s): J. Kerr

Last updated by author(s): 3/13/2020

Reporting Summary

Nature Research wishes to improve the reproducibility of the work that we publish. This form provides structure for consistency and transparency in reporting. For further information on Nature Research policies, see Authors & Referees and the Editorial Policy Checklist .

Statistics

For all statistical analyses, confirm that the following items are present in the figure legend, table legend, main text, or Methods section.

n/a	Confirmed	
☐	☒	The exact sample size (n) for each experimental group/condition, given as a discrete number and unit of measurement
☐	☒	A statement on whether measurements were taken from distinct samples or whether the same sample was measured repeatedly
☐	☒	The statistical test(s) used AND whether they are one- or two-sided<br>*Only common tests should be described solely by name; describe more complex techniques in the Methods section.*
☒	☐	A description of all covariates tested
☒	☐	A description of any assumptions or corrections, such as tests of normality and adjustment for multiple comparisons
☐	☒	A full description of the statistical parameters including central tendency (e.g. means) or other basic estimates (e.g. regression coefficient) AND variation (e.g. standard deviation) or associated estimates of uncertainty (e.g. confidence intervals)
☐	☒	For null hypothesis testing, the test statistic (e.g. F, t, r) with confidence intervals, effect sizes, degrees of freedom and P value noted<br>*Give P values as exact values whenever suitable.*
☒	☐	For Bayesian analysis, information on the choice of priors and Markov chain Monte Carlo settings
☒	☐	For hierarchical and complex designs, identification of the appropriate level for tests and full reporting of outcomes
☒	☐	Estimates of effect sizes (e.g. Cohen's d, Pearson's r), indicating how they were calculated

Our web collection on statistics for biologists contains articles on many of the points above.

Software and code

Policy information about availability of computer code

Data collection	Galvanometric scanning data were acquired using Scanimage version 3.8 and Matlab 2013b. Resonance scanning data were acquired using Scanimage version 5.2 and Matlab 2013b. Visual stimuli were provided by the Psychophysics toolbox operating in Matlab 2013b.
Data analysis	Data analysis was performed using Matlab versions 2013b and 2019a, including the CircStat toolbox version 2012a. Code supporting the figures and analyses in the manuscript available on reasonable request.

For manuscripts utilizing custom algorithms or software that are central to the research but not yet described in published literature, software must be made available to editors/reviewers. We strongly encourage code deposition in a community repository (e.g. GitHub). See the Nature Research guidelines for submitting code & software for further information.

Data

Policy information about availability of data

All manuscripts must include a data availability statement. This statement should provide the following information, where applicable:

- Accession codes, unique identifiers, or web links for publicly available datasets
- A list of figures that have associated raw data
- A description of any restrictions on data availability

Data available on request.

Field-specific reporting

Please select the one below that is the best fit for your research. If you are not sure, read the appropriate sections before making your selection.

☒ Life sciences ☐ Behavioural & social sciences ☐ Ecological, evolutionary & environmental sciences

Life sciences study design

All studies must disclose on these points even when the disclosure is negative.

Sample size	Sample sizes were chosen to minimize the number of experimental animals used while retaining statistical power.
Data exclusions	No data were excluded.
Replication	All experimental findings were repeated in a minimum of 2 animals for anatomical tracing experiments and a minimum of 4 for physiology experiments, all with consistent results. Statements of the number of animals used in each group accompany the relevant statistics provided in the text.
Randomization	No randomization of experimental animals was performed as they were acquired just prior to the commencement of each individual experiment, and the allocation from the supplier is expected to be random.<br>Visual stimuli were presented to the animals in a randomized sequence, including randomization of the eye to which the stimulus was presented.
Blinding	Blinding was not considered relevant during data collection or analysis due to the lack of realistic or relevant methods for the experimenter to influence the experimental outcome, considering the experiments performed, the techniques used and the analyses done.

Reporting for specific materials, systems and methods

We require information from authors about some types of materials, experimental systems and methods used in many studies. Here, indicate whether each material, system or method listed is relevant to your study. If you are not sure if a list item applies to your research, read the appropriate section before selecting a response.

Materials & experimental systems

n/a	Involved in the study	
x		Antibodies
x		Eukaryotic cell lines
x		Palaeontology
	x	Animals and other organisms
x		Human research participants
x		Clinical data

Methods

n/a	Involved in the study	
x		ChIP-seq
x		Flow cytometry
x		MRI-based neuroimaging

Animals and other organisms

Policy information about studies involving animals; ARRIVE guidelines recommended for reporting animal research

Laboratory animals	Rattus norwegicus, Lister hooded, male, weighing 55-137g body weight at the commencement of the experiments.
Wild animals	No wild animals were used in this study.
Field-collected samples	The study did not involve field-collected samples.
Ethics oversight	Ethical oversight was by the Regierungspraesidium Tuebingen and Landesamt für Natur, Umwelt und Verbraucherschutz Nordrhein-Westfalen, Germany.

Note that full information on the approval of the study protocol must also be provided in the manuscript.
